# The mechanism of miR-142-3p in coronary microembolization-induced myocardiac injury via regulating target gene IRAK-1

**DOI:** 10.1038/s41419-019-1341-7

**Published:** 2019-01-25

**Authors:** Qiang Su, Xiangwei Lv, Ziliang Ye, Yuhan Sun, Binghui Kong, Zhenbai Qin, Lang Li

**Affiliations:** 1 Department of Cardiology, The Affiliated Hospital of Guilin Medical University, 15#, Lequn Road, 541001 Guilin, Guangxi China; 2 Department of Cardiology, The First Affiliated Hospital of Guangxi Medical University, 530021 Nanning, China

## Abstract

Coronary microembolization (CME) is a common complication seen during primary percutaneous coronary intervention (pPCI). CME-induced myocardiac inflammation is the primary cause of myocardiac injury. Dysregulated miR-142-3p has been implicated in multiple cardiovascular diseases and is significantly downregulated in CME-induced myocardial injury. However, the role of miR-142-3p in CME-induced myocardial injury is unclear. This study herein built a porcine CME model by infusing microembolization spheres into the left anterior descending branch via a microcatheter, and detected the downregulation of miR-142-3p in the myocardial tissues of CME pigs. Echocardiography, hematoxylin basic fuchsin picric acid (HBFP) staining, and western blotting of NF-κB p65, TNF-α, IL-1β, and IL-6 showed that the pharmacological overexpression of miR-142-3p using agomiR has improved cardiac function and attenuated CME-induced myocardiac inflammatory response, while its inhibition using antagomiR demonstrated inverse effects. Moreover, in vitro experiments demonstrated IRAK-1 as a direct target gene of miR-142-3p. Luciferase reporter assays, quantitative real-time polymerase chain reaction and western blotting demonstrated its effects in controlling the inflammation of cardiomyocytes. It is noteworthy that miR-142-3p was found to be decreased in the plasma of STEMI patients undergoing pPCI with no-reflow, indicating a potential clinical relevance of miR-142-3p. The receiver–operator characteristic curve indicated that plasma miR-142-3p might be an independent predictor of no-reflow during pPCI in patients with STEMI. Therefore, overexpression of miR-142-3p acts as a novel therapy for CME-induced myocardial injury.

## Introduction

Coronary microembolization (CME) is a common complication seen during the emergency treatment of acute myocardial infarction (AMI) by primary percutaneous coronary intervention (pPCI), with an incidence rate of 15–20%^1^. CME can directly cause the no-reflow or slow-reflow phenomenon, and is considered as an independent predictor for long-term adverse prognosis and the incidence of primary heart adverse events of AMI^2,3^. Previous studies have demonstrated that there were several inflammatory cells infiltrating from the peri-foci area of CME-induced myocardiac microinfarction, and is accompanied by excessive release of inflammatory factors. This in turn elicits local myocardial inflammatory response, and remains the key element that leads to post-CME myocaridal injury and progressive cardiac dysfunction^4,5^. Li et al. further uncovered that extensive NF-κB activation results in the excessive release of inflammatory mediators (such as TNF-α and IL-1β), which played an important role in CME-induced progressive cardiac dysfunction and advanced heart failure. While CME-induced local myocardiac inflammatory response was prominently alleviated and the cardiac function was markedly improved after NF-κB activity was suppressed by a specific inhibitor PDTC^6^. Therefore, NF-κB-signaling pathway activation that results in the excessive release of varied inflammatory mediators plays a critical role in CME-induced myocardial injury. Nonetheless, the specific gene regulatory principle and molecular mechanisms remain unclear.

MicroRNAs (miRNAs, miRs) are endogenous non-coding small molecule RNAs, and are about 21–25 nucleotides in length. These universally exist in animals, plants, viruses, and single-cell organisms, and bind to target mRNA 3′ non-coding (3′UTR) region via complete/incomplete complementary binding. This subsequently suppresses the target mRNA translation or promotes its degradation, regulating the target gene expression at the posttranslational level^7,8^. Our previous study has reported the existence of differential expression mRNAs in the myocardial tissue of CME pigs, and showed significant downregulation of miR-142-3p in comparison to the sham operation group^9^. The dysregulation of miR-142-3p has recently been reported to play an important role in multiple cardiovascular diseases like myocardial ischemia-reperfusion injury and diabetic cardiomyopathy^10,11^. However, the functional and molecular mechanisms of myocardial miRNA dysregulation in CME-induced myocardial injury are largely unknown.

Hence, this study herein built a porcine CME model by infusing microembolization spheres into the left anterior descending branch via microcatheter. Results demonstrated that pre-treatment with miR-142-3p mimics before CME modeling significantly improved cardiac functions, while the inflammatory factors TNF-α and IL-1β were markedly reduced in the myocardial tissue. Furthermore, pre-treatment with miR-142-3p inhibitor before CME modeling aggravated cardiac dysfunction, while the inflammatory factors TNF-α and IL-1β were further elevated in the myocardial tissue. Additionally, IRAK-1 was identified as a novel target gene of miR-142-3p. Finally, the clinical relevance of miR-142-3p was confirmed by its decrease in the plasma of ST-segment elevation myocardial infarction (STEMI) patients undergoing pPCI with no-reflow when compared to STEMI patients undergoing pPCI without no-reflow. Collectively, our data provided strong evidence that miR-142-3p controls CME-induced myocardial injury via regulating IRAK-1. Increased expression of miR-142-3p might act as a protective strategy for treating CME-induced myocardial injury.

## Results

### Downregulation of miR-142-3p in the myocardiac tissue of CME pigs

**Fig. 1:**
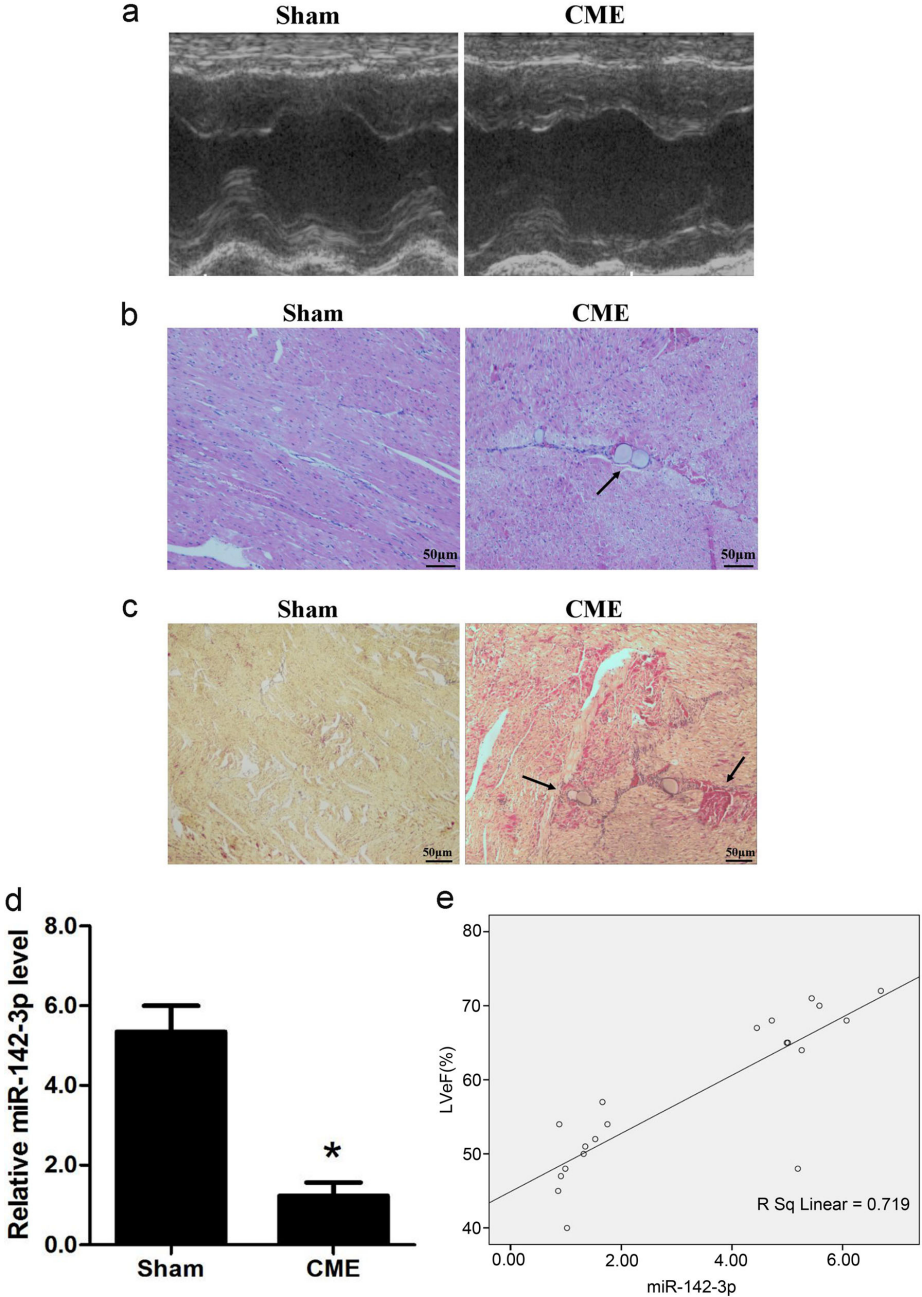
Downregulation of miR-142-3p in the myocardiac tissue of CME pig. **a** Echocardiographic analysis of Sham and CME pigs. Left ventricle ejection fraction (LVEF, %), left ventricle fractional shortening (LVFS, %), cardiac output (CO, L/min) and left ventricular end-diastolic diameter (LVEDd, mm) were quantitatively analyzed (*n* = 10). **b** Hematoxylin and eosin (H&E) staining of local micro-infracted lesions in the myocardium in the CME group. Arrow indicates microspheres (×400) (*n* = 10). **c** Hematoxylin-basic fuchsin-picric acid (HBFP) staining of samples from the Sham, and CME groups. Ischemic myocardium appeared in red. Arrow indicated a micro-infracted lesion (×200) (*n* = 10). **d** Relative expression of miR-142-3p was determined by quantitative real-time polymerase chain reaction (qRT-PCR) in the heart (*n* = 10). **e** The correlation between miR-142-3p levels and LVEF (%) (*n* = 10). ^*^P < 0.05

As described in the sections method, the porcine model of CME was established by infusing microembolization sphere into the left anterior descending branch via microcatheter. Compared with sham group, CME group showed a significant reduction in cardiac functions, as reflected by marked reductions of LVEF, FS, and CO, while a rise in LVEDd (*P* < 0.05, Table 1 and Fig. 1a). Meanwhile, serum c-troponin I content was significantly higher in CME group than sham group, 0.235 ± 0.031 versus 0.037 ± 0.006 ng/ml, respectively (*P* *<* 0.05). As shown in Fig. 1b, HE staining of CME group displayed nuclear lysis or absence and red colored cytoplasm in the myocardiac cells of microembolization foci, while the adjacent myocardium demonstrated edema and degeneration, and the peri-infarction area was infiltrated with inflammatory cells and red blood cells. Hematoxylin basic fuchsin picric acid (HBFP) staining in sham operation group displayed occasional subendocardial ischemia, without any obvious signs of infarction foci. As shown in Fig. 1c, under optical microscope, microembolization spheres were found in the arterioles of CME group, accompanied by adjacent microinfarction foci that are mostly wedge-shaped and locally distributed. qRT-PCR results demonstrated a significant reduction in miR-142-3p in CME group (Fig. 1d), and correlation analysis demonstrated positive correlation with GBM pig cardiac function LVEF (Fig. 1e).

**Table 1:** Cardiac function measured by echocardiography

Group	*n*	LVEF (%)	LVFS (%)	CO (l/min)	LVEDd (mm)
Sham	10	69.38 ± 2.77	42.04 ± 5.88	3.74 ± 0.75	3.27 ± 0.39
CME	10	48.82 ± 3.68^*^	26.41 ± 7.56^*^	2.76 ± 0.84^*^	4.97 ± 0.55^*^

*NC* negative control, *LVEF* left ventricle ejection fraction, *CO* cardiac output, *LVEDd* left ventricular end-diastolic diameter, *LVFS* left ventricle fractional shortening, *CME* coronary microembolization

^*^P   < 0.05 Sham versus CME

### miR-142-3p agomiR improves cardiac function and attenuates CME-caused myocardiac inflammatory response

**Fig. 2:**
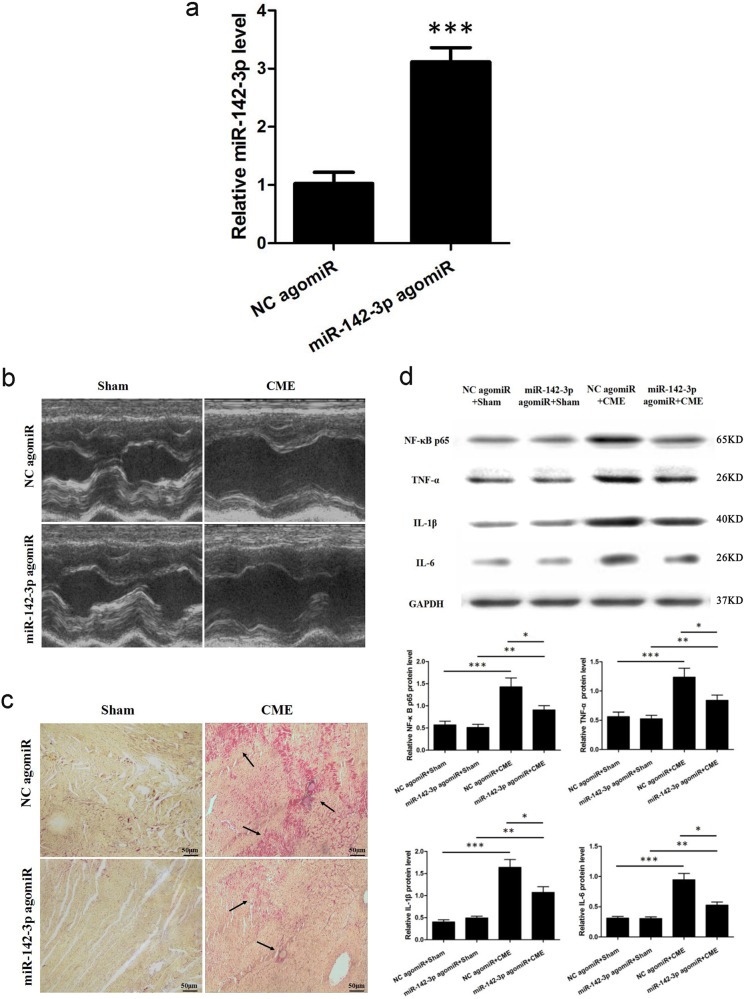
miR-142-3p agomiR improves cardiac function and attenuates CME-caused myocardiac inflammatory response. **a** Relative miR-142-3p expression level was increased in the hearts of pigs after miR-142-3p agomiR treatment as determined by quantitative real-time polymerase chain reaction (qRT-PCR) (*n* = 5). **b** Left ventricle ejection fraction (LVEF, %), left ventricle fractional shortening (LVFS, %), cardiac output (CO, l/min) and left ventricular end-diastolic diameter (LVEDd, mm) were measured by echocardiography (*n* = 5). **c** Hematoxylin-basic fuchsin-picric acid (HBFP) staining of samples from the negative control (NC) agomiR + Sham, miR-142-3p agomiR + Sham, NC agomiR + CME, and miR-142-3p agomiR + CME groups, respectively. Ischemic myocardium appeared in red. Arrow indicates a micro-infracted lesion (×200) (*n* = 5). **d** Western blot detection and quantification were performed for NF-κB p65, TNF-α, IL-1β, and IL-6. GAPDH was used as an internal control (*n* = 5). ^*^P < 0.05; **P < 0.01; ^***^P < 0.001

To further explore the effect of miR-142-3p on CME, 400 μl of miR-142-3p agomiR transfection complex was infused via the microcatheter into the left anterior descending branch at 72 h ahead of CME modeling. miR-142-3p was significantly elevated (Fig. 2a). miR-142-3p agomiR prominently improved CME-caused cardiac dysfunctions (Table 2 and Fig. 2b), while serum c-troponin I content was lower than in NC agomiR + CME group, 0.107 ± 0.013 versus 0.242 ± 0.033 ng/ml, respectively (*P* *<* 0.05). Moreover, HBFP staining showed less microinfarction area compared to NC agomiR + CME group, (8.12 ± 2.93)% versus (14.16 ± 3.67)%, (*P* *<* 0.05; Fig. 2c). Western blotting results demonstrated a significant reduction in the levels of NF-κB p65, TNF-α, IL-1β, and IL-6 compared with those in the CME group (Fig. 2d). These results suggested that miR-142-3p agomiR could alleviate CME-caused myocardiac injury and attenuate CME-induced myocardiac inflammatory response.

**Table 2:** Cardiac function measured by echocardiography with miR-142-3p agomiR

Group	*n*	LVEF (%)	LVFS (%)	CO (l/min)	LVEDd (mm)
NC agomiR + Sham	5	70.63 ± 2.75	42.07 ± 5.98	3.46 ± 0.89	3.14 ± 0.46
miR-142-3p agomiR + Sham	5	69.24 ± 2.86	40.89 ± 5.61	3.38 ± 0.93	3.37 ± 0.53
NC agomiR + CME	5	44.69 ± 3.62^*^	23.72 ± 7.35^*^	2.55 ± 0.79^*^	5.47 ± 0.62^*^
miR-142-3p agomiR + CME	5	53.34 ± 3.41^**,***^	29.91 ± 7.64^**,***^	2.93 ± 0.81^**,***^	4.78 ± 0.58^**,***^

*NC* negative control, *LVEF* left ventricle ejection fraction, *CO* cardiac output, *LVEDd* left ventricular end-diastolic diameter, *LVFS* left ventricle fractional shortening, *CME* coronary microembolization

^*^P   < 0.05 NC agomiR  + Sham versus NC agomiR  +  CME; ^**^P   < 0.05 miR-142-3p agomiR  +  Sham versus miR-142-3p agomiR  +  CME; ^***^P  <  0.05 NC agomiR  +  CME versus miR-142-3p agomiR  +  CME

### miR-142-3p inhibition aggravates CME-caused myocardiac injury and CME-induced myocardiac inflammatory response

**Fig. 3:**
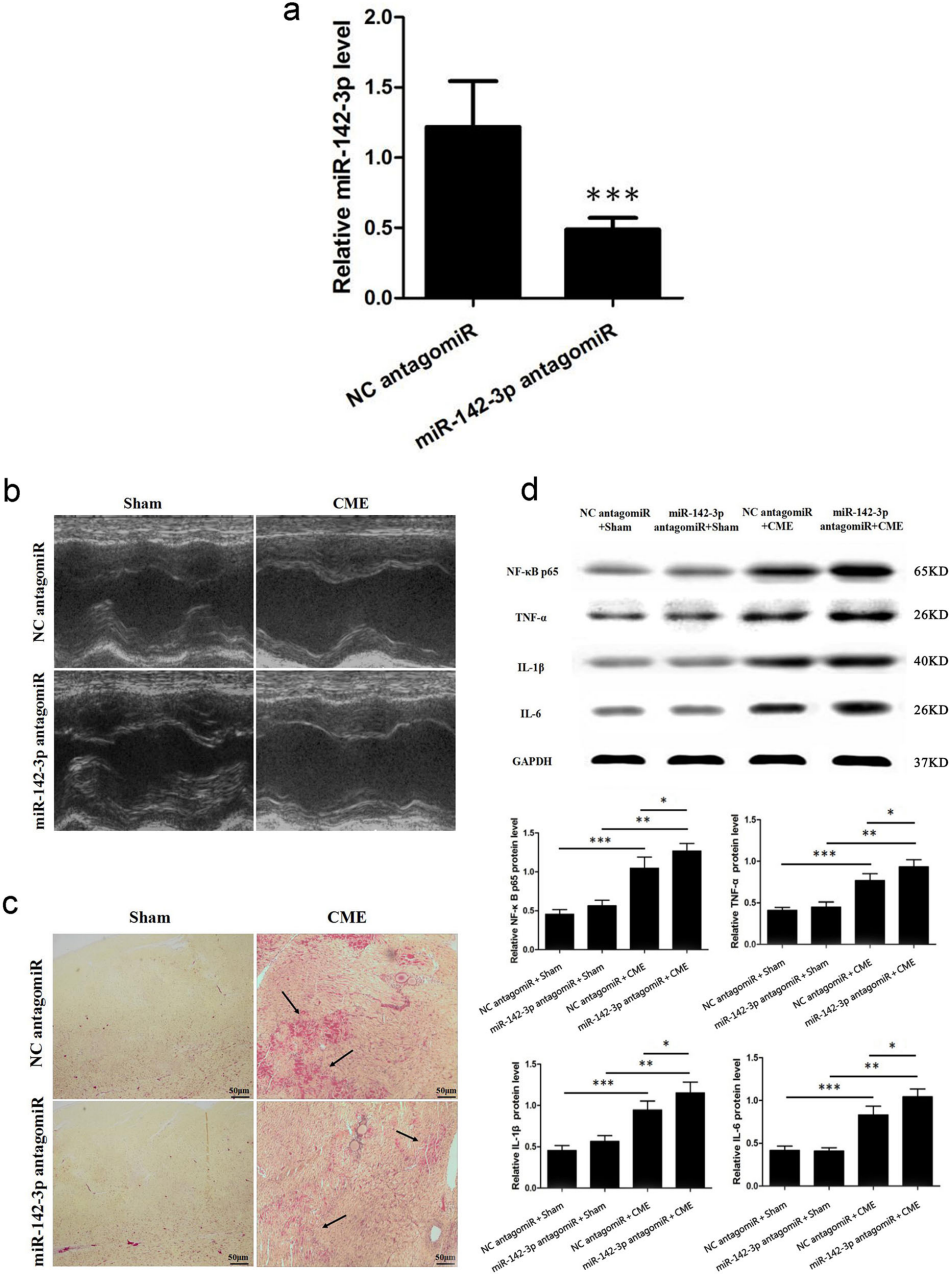
miR-142-3p inhibition aggravates CME-caused myocardiac injury and CME-induced myocardiac inflammatory response. **a** Relative miR-142-3p expression level was decreased in hearts of pig after miR-142-3p antagomiR treatment as determined by quantitative real time-polymerase chain reaction (qRT-PCR) (*n* = 5). **b** Left ventricle ejection fraction (LVEF, %), left ventricle fractional shortening (LVFS, %), cardiac output (CO, l/min) and left ventricular end-diastolic diameter (LVEDd, mm) were measured by echocardiography (*n* = 5). **c** Hematoxylin-basic fuchsin-picric acid (HBFP) staining of samples from the negative control (NC) antgomiR + Sham, miR-142-3p antagomiR + Sham, NC antagomiR + CME, and miR-142-3p antagomiR + CME groups, respectively. Ischemic myocardium appeared in red. Arrow indicates a micro-infracted lesion (×200) (*n* = 5). **d** Western blot detection and quantification of NF-κB p65, TNF-α, IL-1β and IL-6. GAPDH was used as an internal control (*n* = 5). ^*^P < 0.05; ^**^P < 0.01; ^***^P < 0.001

Likewise, 400 μl miR-142-3p antagomiR transfection complex was infused via microcatheter into the left anterior descending branch at 72 h ahead of CME modeling. miR-142-3p content was significantly declined (Fig. 3a). miR-142-3p antagomiR significantly aggravated CME-caused cardiac dysfunction (Table 3 and Fig. 3b), while raised the serum c-troponin I content compared with that in the CME group, 0.326 ± 0.027 versus 0.201 ± 0.018 ng/ml, respectively (*P* *<* 0.05). Moreover, HBFP staining revealed an increase in the microinfarction area compared with that in the CME group, (19.38 ± 4.64)% versus (13.71 ± 3.56)% (*P* *<* 0.05; Fig. 3c). Western blotting results demonstrated a significant rise in the NF-κB p65, TNF-α, IL-1β, and IL-6 levels compared with those in the CME group (Fig. 3d). These results suggest that miR-142-3p antagomiR aggravated CME-caused myocardiac injury and enhanced CME-induced myocardiac inflammatory response.

**Table 3:** Cardiac function by echocardiography with miR-142-3p antagomiR

Group	*n*	LVEF (%)	LVFS (%)	CO (l/min)	LVEDd (mm)
NC antagomiR + Sham	5	70.88 ± 2.81	42.69 ± 6.02	3.47 ± 0.86	3.18 ± 0.45
miR-142-3p antagomiR + Sham	5	67.56 ± 2.87	40.03 ± 5.65	3.32 ± 0.94	3.42 ± 0.52
NC antagomiR + CME	5	47.24 ± 3.47^*^	25.08 ± 7.24^*^	2.63 ± 0.77^*^	5.41 ± 0.59^*^
miR-142-3p antagomiR + CME	5	36.85 ± 3.96^**,***^	19.94 ± 6.38^**,***^	2.27 ± 0.69^**,***^	6.17 ± 0.64^**,***^

*NC* negative control, *LVEF* left ventricle ejection fraction, *CO* cardiac output, *LVEDd* left ventricular end-diastolic diameter, *LVFS* left ventricle fractional shortening, *CME* coronary microembolization

^*^P   < 0.05 NC antagomiR  +  Sham versus NC antagomiR + CME; ^**^P   < 0.05 miR-142-3p antagomiR  +  Sham versus miR-142-3p antagomiR + CME; ^***^P   < 0.05 NC antagomiR  +  CME versus miR-142-3p antagomiR + CME

### IRAK-1 is a target gene of miR-142-3p

**Fig. 4:**
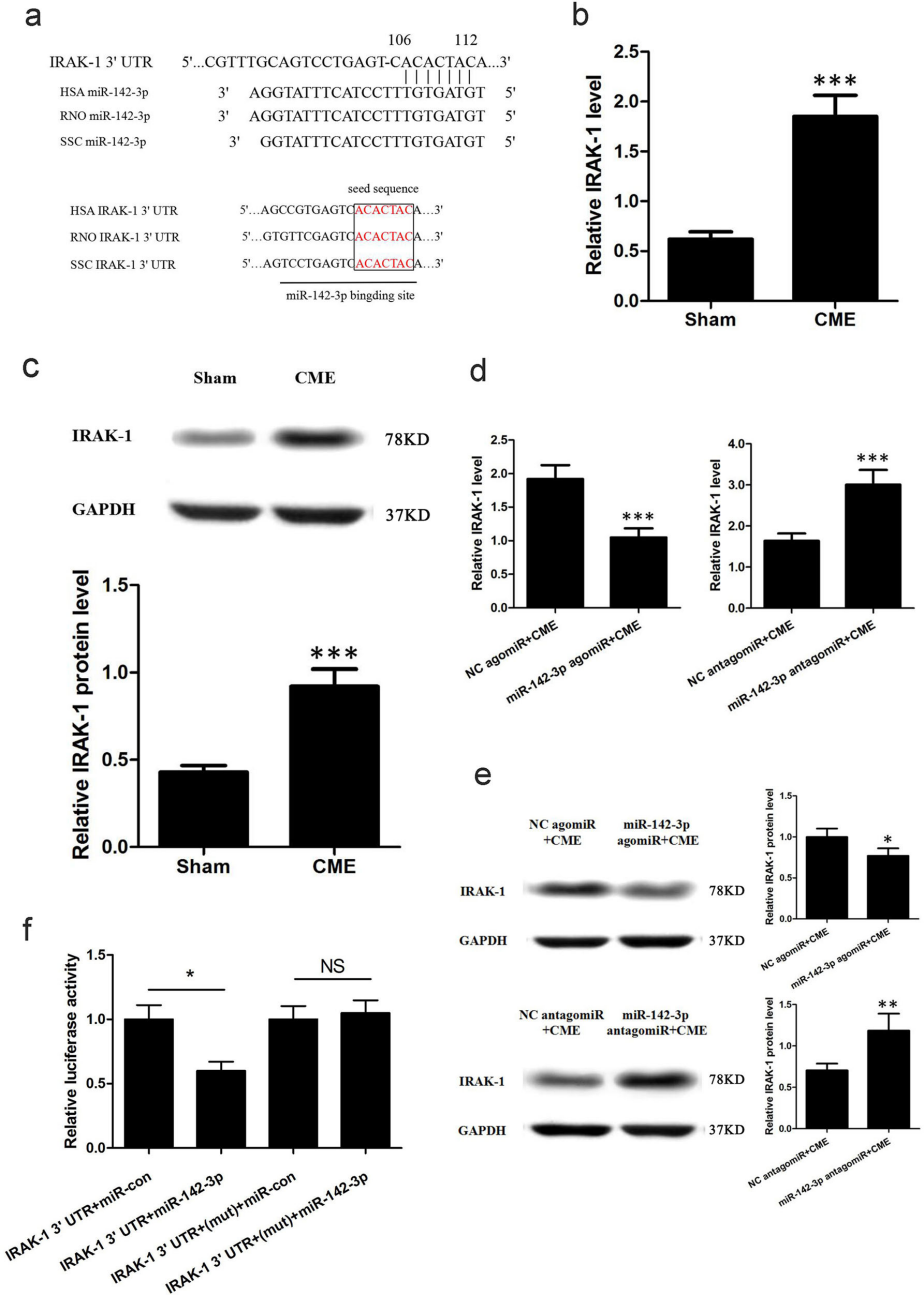
miR-142-3p directly targets IRAK-1. **a** Sequence alignment of miR-142-3p and IRAK-1 3′UTR from different species. **b** Quantitative real-time polymerase chain reaction (qRT-PCR) was performed to determine the expression of IRAK-1 (*n* = 10). **c** Western blot detection and quantification for IRAK-1.G APDH was used as an internal control (*n* = 10). **d** IRAK-1 was inversely regulated by miR-142-3p at the mRNA level in heart tissues (*n* = 5). **e** IRAK-1 was inversely regulated by miR-142-3p at the protein level in heart tissues. GAPDH was used as an internal control (*n* = 5). **f** HEK293 cells were transfected with wild-type or mutated IRAK-1 3′UTR together with miR-142-3p agomiR or miRNA negative control (*n* = 3). ^*^P < 0.05; ^**^P < 0.01; ^***^P < 0.001

We first used a bioinformatics approach using TargetScan and miRBase to predict the target genes of miR-142-3p. Notably, IRAK-1 was identified as a potential miR-142-3p target (Fig. 4a). By aligning human, rat, and pig IRAK-1 3′UTRs, we noted that the predicted miR-142-3p-binding sites were highly conserved, suggesting that these sites may be important for miR-142-3p regulation of IRAK-1 expression (Fig. 4a). Additionally, predicted miR-142-3p target gene IRAK-1, either at the gene or protein level, was significantly elevated in CME group (*P* < 0.05; Fig. 4b, c). Moreover, at both mRNA and protein levels, IRAK-1 was downregulated in miR-142-3p agomiR-treated pigs, while upregulated in miR-142-3p antagomiR-treated pigs, suggesting an inverse correlation between IRAK-1 and miR-142-3p expression (Fig. 4d, e). Luciferase reporter assay revealed that compared with negative control (NC), the luciferase activity was decreased by miR-142-3p agomiR and if the predicted miR-142-3p-binding site on miR-142-3p 3′UTR was mutated, the binding of miR-142-3p to IRAK-1 3′UTR was prevented and the decrease in luciferase activity was abrogated. These results indicated that IRAK-1 acts as a direct target gene of IRAK-1 (Fig. 4f).

### Up-regulation of miR-142-3p and silencing of IRAK-1 decreased the levels of NF-κB p65, TNF-α, IL-1β, and IL-6 in cardiomyocytes

**Fig. 5:**
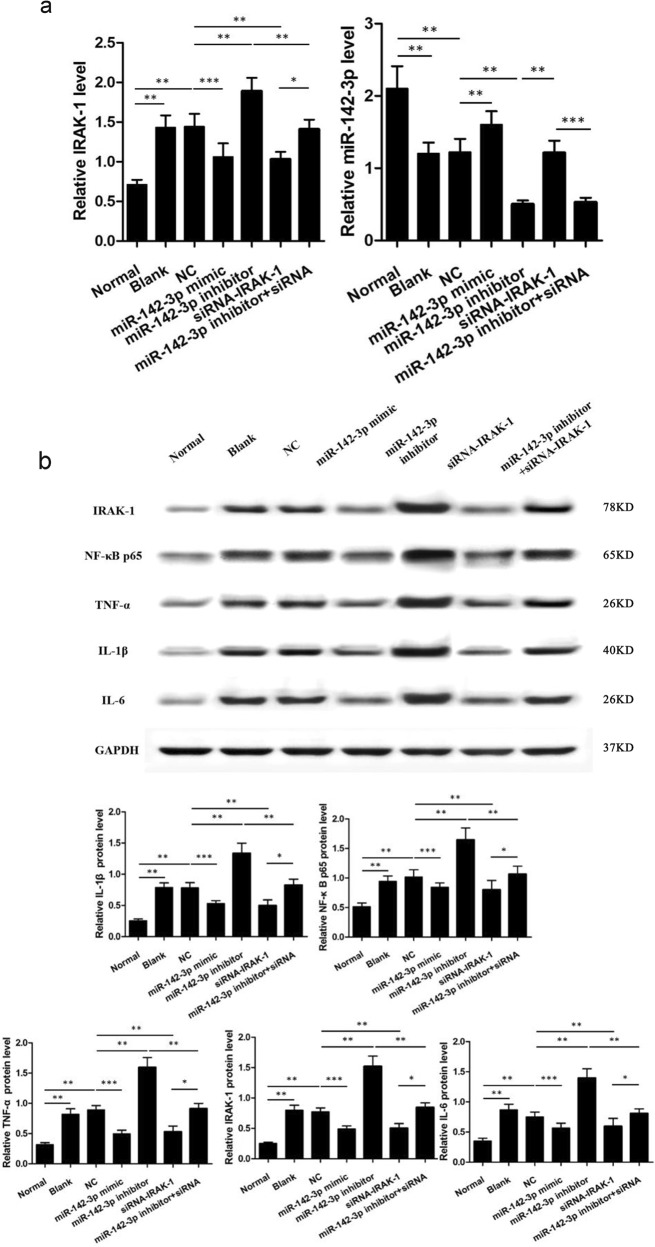
IRAK-1 mediates the effects of miR-142-3p on inflammation of cardiomyocytes. **a** Quantitative real-time polymerase chain reaction (qRT-PCR) was performed to determine the expression of miR-142-3p and IRAK-1 (*n* = 4). **b** Western blot and quantification were done for determining the IRAK-1, NF-κB p65, TNF-α, IL-1β, and IL-6 expressions. GAPDH was used as an internal control (*n* = 4). ^*^P < 0.05; ^**^P < 0.01; ^***^P < 0.001

qRT-PCR and western blotting results demonstrated that all other groups have elevated contents of IRAK-1, NF-κB p65, TNF-α, IL-1β, and IL-6 compared with those in the normal group (all *P* < 0.05). These parameters showed no significant differences between blank and NC groups (*P* > 0.05). Compared with blank and NC groups, the levels of IRAK-1, NF-κB p65, TNF-α, IL-1β, and IL-6 were decreased in the miR-142-3p mimic and the siRNA-IRAK-1 groups, but increased in the miR-142-3p inhibitor group (all *P* < 0.05). There was no significant difference between the miR-142-3p inhibitor + siRNA-IRAK-1 group and the blank and the NC groups (Fig. 5a, b).

### Decreased miR-142-3p level in STEMI patients undergoing pPCI with no-reflow

**Fig. 6:**
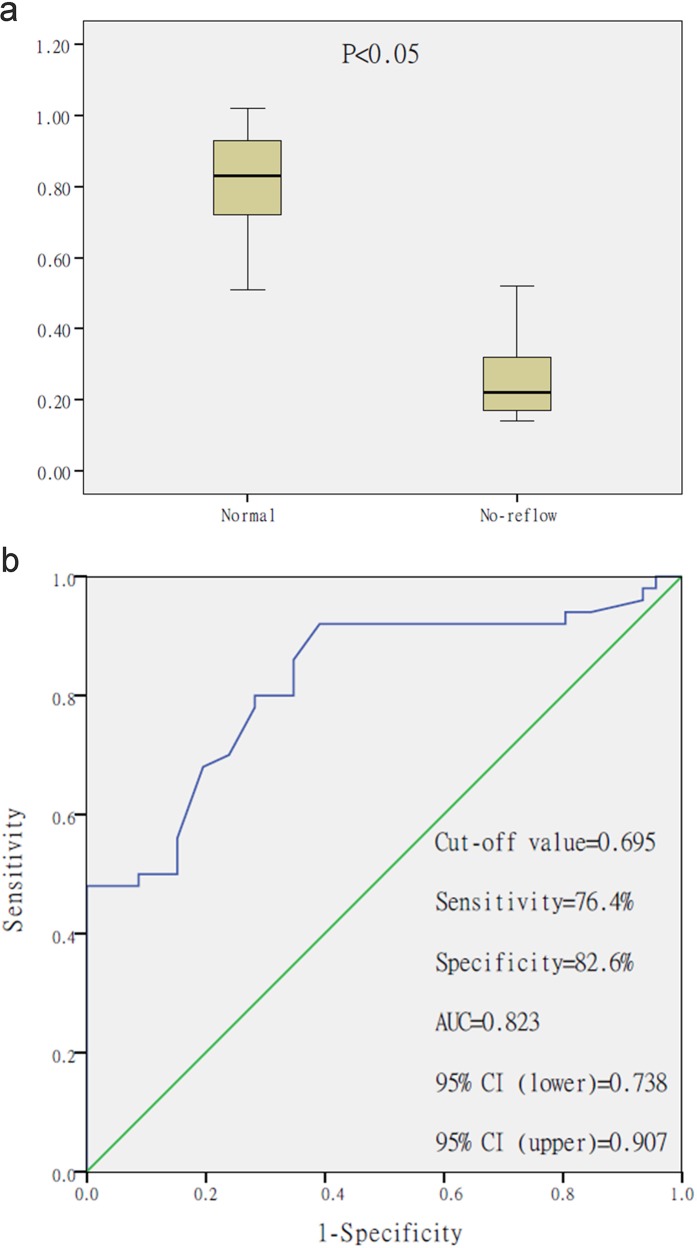
Decreased plasma miR-142-3p level in STEMI patients undergoing pPCI with no-reflow. **a** Quantitative real-time polymerase chain reaction (qRT-PCR) showed that miR-142-3p expression level was decreased in the plasma of STEMI patients undergoing pPCI without no-reflow (*n* = 214) when compared with STEMI patients undergoing pPCI with no-reflow (*n* = 41). **b** The receiver–operator characteristic curve for the plasma miR-142-3p acts as a potential biomarker for STEMI patients undergoing pPCI with no-reflow

The plasma level of miR-142-3p was examined in a total of 41 STEMI patients undergoing pPCI with no-reflow versus 214 STEMI patients undergoing pPCI without no-reflow. It is noteworthy that miR-142-3p showed marked decrease in the plasma of STEMI patients undergoing pPCI with no-reflow (Fig. 6a). This suggested that the lower miR-142-3p levels during admission are associated with no-reflow in STEMI patients undergoing pPCI. The clinical characteristics of these patients were reported in our previous study^12^. The receiver– operator characteristic (ROC) curve analysis indicated that plasma miR-142-3p might be a biomarker for STEMI patients undergoing pPCI with no-reflow with an area under the curve of 0.823 (95% confidence interval: 0.738–0.907, *P* < 0.05) (Fig. 6b).

## Discussion

CME is a clinically common and intractable complication seen in acute coronary syndrome (ACS), ischemic heart disease, PCI, and thrombolytic therapy. It is also considered as an important cause of coronary microcirculation dysfunction^13^. The occurrence of CME could directly cause coronary no-reflow or slow-reflow, and could elicit a variety of common complications, including arrhythmia, decreased coronary fractional flow reserve (FFR), insufficient myocardial perfusion, myocardial microinfarction, and myocardial systolic dysfunction^14–16^. Our previous study has detected the role of different miRNAs, including miR-92b-5p, miR-491, miR-874, miR-425-3p, miR-376a-5p, miR-370, miR-30c-3p, miR-493-5p, miR-323, miR-136, and miR-142-3p in the myocardiac tissue of porcine CME model. After validation, miR-142-3p was significantly downregulated in the myocardiac tissue of CME pigs, with the most prominent alteration^9^. miR-142-3p remain a critical miRNA that regulates inflammation, and play important roles in multiple cardiovascular diseases like myocardial ischemia-reperfusion injury and diabetic cardiomyopathy^10,11^. However, the role of miR-142-3p in CME-induced myocardial dysfunction is still unclear. The key findings of our study are as follows: (1) miR-142-3p was markedly declined in CME pig, while its overexpression prevented cardiac dysfunction and attenuated myocardiac inflammatory response; (2) IRAK-1 might act as a target gene of miR-142-3p; (3) miR-142-3p was decreased in the plasma of STEMI patients undergoing pPCI with no-reflow and might be an independent predictor of no-reflow during pPCI in patients with STEMI.

As reported in the literature, miR-142-3p plays a role in varied inflammatory diseases, such as atherosclerosis^17^, heart transplantation^18^, sepsis^19^, and systemic lupus erythematosus^20^. However, the specific function of miR-142-3p and its regulatory mechanism have been rarely probed. miR-142-3p expression in the myocardial tissue of CME pigs was abnormal, which indicated its role in the pathogenesis and development of CME-induced myocardiac inflammation. To better delineate the effect of miR-142-3p on CME-induced myocardiac injury, our study herein applied pPCI to build CME model via infusing microspheres into the coronary artery. Moreover, in vitro inflammatory model was built in the primary neonatal rat cardiacmyocytes by inducing with LPS. Our findings revealed low expression of miR-142-3p both in in vitro and in vivo systems, indicating the involvement of miR-142-3p in CME-induced myocardiac inflammatory response.

Several studies have reported transient decrease of coronary flow when CME could be instantly resumed to normal, whereas the systolic function of local myocardium was deteriorated progressively. CME-induced myocardial injury was not completely caused by the reduction in coronary flow, while local myocardial inflammatory response was indeed the primary reason for the progression of systolic dysfunction^21,22^. More studies uncovered the role of TLR4/NF-κB signaling pathway in post-CME myocardial immune-inflammatory response, and this activated NLRP3 inflammasome, thereby promoting inflammation cascades and aggravating myocardiac injury. Inhibition of this signaling pathway effectively alleviated CME-induced local myocardial inflammatory response and markedly improved the cardiac functions^23,24^. In these regards, activation of TLR4/NF-κB-signaling pathway showed excessive release of varied inflammatory mediators, and played a critical role in CME-induced myocardiac injury. Nonetheless, the specific gene regulatory principle and molecular mechanism yet remained unclear. Interestingly, our study herein deployed pre-treatment with miR-142-3p agomiR prior to CME operation, which subsequently decreased the inflammatory factors in the myocardium, and then suppressed the NF-κB- signaling pathway. In contrast, when pre-treatment with miR-142-3p antagomiR was deployed, NF-κB-signaling pathway was prominently activated, which then aggravated CME-induced myocardiac inflammatory response and cardiac injury. These data indicated an important role of miR-142-3p in CME-induced myocaridal inflammatory response. miR-142-3p overexpression suppressed CME-induced myocardial inflammatory response, which could be enacted via alleviating NF-κB-signaling pathway by excessive immunoinflammation in the myocardium.

According to a study, miR-142-3p via targeted inhibition of IRAK1 expression was involved in the regulation of TLR4/MyD88-signaling pathway. Hence, modulating the expression of NF-κB downstream inflammatory factors TNF-α and IL-6 to prevent excessive inflammation and immune response in the human body^25^. Interleukin-1 receptor-associated kinase (IRAK) family is a specific serine/threonine kinase, and the identified IRAKs include IRAK1, IRAK2, IRAK-M, and IRAK4. IRAK1 is an element that is involved in toll-like receptor (TLR)-signaling pathway, and plays an important connective and pivotal role in modulating the immunity^26^. Several studies have reported that multiple specific miRNAs modulated inflammatory response via targeting IRAK1. miR-K9 was found to directly target IRAK1, thereby regulating endothelial cell inflammatory response^27^. miR-146a by targeting the regulation of IRAK1 expression was involved in the inflammatory response of gliocytes^28^. Our study demonstrated that miR-142-3p directly targeted IRAK13′-UTR, and miR-142-3p mimics transfection by significantly suppressing IRAK1 expression, inhibiting secretion of inflammatory factors. Moreover, IRAK-1 expression in the myocardium was evidently elevated after CME modeling, indicating that miR-142-3p was involved in CME-induced myocardial inflammatory response via regulating its target gene IRAK-1. To further elucidate the role of miR-142-3p in cardiac myocytes inflammatory response, LPS was applied to induce in vitro inflammatory response and IRAK-1/NF-κB-signaling pathway activation in primary neonatal rat cardiacmyocytes. Results demonstrated that miR-142-3p overexpression by vector transfection prominently alleviated cardiac myocytes inflammatory response and suppressed IRAK-1/NF-κB-signaling pathway, which was considered relevant for the downregulation of its target gene IRAK1. These data indicated that IRAK1 was the downstream target gene of miR-142-3p and was involved in CME-induced myocardial inflammatory response.

It is noteworthy that we detected a remarkable decrease in the circulating miR-142-3p levels in STEMI patients undergoing pPCI with no-reflow, and this suggested clinical relevance of miR-142-3p. The ROC curve showed that the plasma miR-142-3p levels were independent predictors of no-reflow during pPCI in patients with STEMI.

However, our study has several limitations. Firstly, the microembolization reagent used for CME modeling was a type of plastic microspheres. Despite harboring the physical characteristics of microvessel embolization, these spheres lacked biological functions (such as thrombus activity, vasoactive activity, chemotaxis, and inflammation). But the constituents of clinical microvessel thrombus are very complicated, which comprised of platelets, white and red blood cells, and components of atheromatous plaque. Therefore, our model varied to a certain extent from clinical CME pathophysiological changes caused by atheromatous plaque rupture. Secondly, more clinical plasma samples of patients are required to confirm if miR-142-3p could act as a biomarker for no-reflow of coronary artery in STEMI patients undergoing pPCI.

In conclusion, this study showed that miR-142-3p is decreased in CME-induced myocardial injury. Pharmacological overexpression of miR-142-3p improved cardiac function and reduced inflammation in the hearts with CME. The miR-142-3p/IRAK-1 pathway might act as a potential and novel therapeutic target for CME-induced myocardial injury.

## Materials and methods

### Animals and CME-induced myocardial injury

Healthy Guangxi Bama Mini-pigs (GBM pigs), male or female, with a body weight of 25–30  kg, were provided by Animal Science and Technology School, Guangxi University. The CME model was established by the following procedure. Basal anesthesia was administered by intramuscular injection of ketamine hydrochloride injection (5–10 mg/kg). After 3–5 min, the experimental pig remained unstable in standing position, and was rinsed after lying on the floor prior to routine disinfection and sterile surgical towels placement. Next, 5½ gauge scalp needle was used to puncture the ear vein and placed an intravenous infusion of diazepam (0.5 mg/kg/h) as a maintenance anesthesia. Then, the unilateral femoral artery was separated and punctured, prior to 6 F arterial sheath implantation, through which heparin (200 U/kg) was infused until heparinized, followed by heparin maintenance (100 U/kg/h). After that, coronary artery angiography was performed, and a microcatheter was inserted via a guiding catheter to the distal end of the first diagonal branch of the left anterior descending coronary artery by infusing microembolization spheres (0.1 million of 42 μm spheres in 1.5 ml physical saline solution) (Biosphere Medical Inc., USA) to build the porcine model of CME^29,30^. Likewise, the sham operation group was infused with 1.5 ml physical saline solution. At the indicated time points after CME or saline treatment, pigs were harvested and heart samples were fixed in 4% paraformaldehyde (PFA) or snap frozen in liquid nitrite and stored at −80 °C for further analysis. This study was approved by the ethics committees of Guangxi Medical University and all animal experiments were conducted under the established guidelines on the use and care of laboratory animals for biomedical research published by National Institutes of Health (No. 85-23, revised 1996).

### miR-142-3p agomiR or antagomiR treatment in pigs

miR-142-3p agomiR and antagomiR were synthesized by GenePharma (Shanghai, China). To examine the functional role of miR-142-3p in CME-induced myocardial injury, miR-142-3p agomiR (a 2′OME + 5′chol-modified miR-142-3p agonist, GenePharma) or antagomiR (a 2′OME + 5′chol-modified miR-142-3p inhibitor, GenePharma) was used to regulate the expression level of miR-142-3p in pigs.

In brief, we first prepared a nucleic acid dilution by dissolving 100 µg of miR-142-3p agomiR (antagomir) or NC agomiR (antagomir) in 100 µl of a sterilized double-distilled H_2_O. Then, we added 100 µl of sterile 10% glucose solution and mixed the solution well. Next, we prepared a transfection reagent dilution by dissolving 50 µl of transfection reagent dilution of transfection reagent in 100 µl of sterile 10% glucose solution and in 50 µl of sterilized doubledistilled H_2_O, and the solution was mixed well. Finally, the nucleic acid dilution and transfection reagent dilution were mixed (1:1) to form a working solution. The mixtures were incubated for 15 min at room temperature for intracoronary injection. At 72 h before CME/sham modeling, 400 μl miR-142-3p agomiR/antagomir transfection complex or NC agomiR/antagomir transfection complex was infused via microcatheter into the left anterior descending branch. The effects of miR-142-3p agomiR or antagomiR treatment were confirmed by measuring the miR-142-3p expression level in the myocardium using quantitative real-time polymerase chain reaction (qRT-PCR).

### Detection of GBM pig cardiac function (Philips sonos7500 series, USA)

Our previous study has revealed the worst cardiac function within 9 h after CME modeling. Therefore, 9 h post CME was chosen as the inspection time point. The following parameters were measured in the two GBM pig groups: left ventricular ejection fraction (LVEF), left ventricular end-diastolic diameter (LVEDd), left ventricle fractional shortening (FS), and cardiac output (CO). The probe frequency was 12 MHz. All values were the average measurements of three cardiac cycles^31^. Echocardiographic data were recorded and analyzed by blinding to different treatments.

### Measurement of serum c-troponin I

Blood (1.0 ml) was obtained from the femoral vein of each pig at 9 h after Sham operation or CME prior to sacrifice. Serum c-troponin I was detected by electrochemistry according to the manufacturer’s instructions (Roche, Inc., Switzerland).

### Measurement of myocardial microinfarct size

Under anesthesia, animals were sacrificed by injecting 10% potassium chloride into the ear vein. The chest was then opened to remove the heart, followed by cutting the ventricle into six slices (5–8 mm in thickness) parallely to the atrioventricular sulcus. Next, 300 mg myocardiac tissue from the anterior wall of the left ventricle was fixed in 4% PFA before proceeding to pathological sectioning for microinfarction detection by HE staining and HBFP staining. HBFP staining is an important method for diagnosing early myocardial ischemia, wherein the ischemic myocardium and red blood cells are in red color, while the cytoplasm of normal myocardial cells was in yellow color and their nucleus in blue color. DMR + Q550 pathological image analyzer was used to analyze the infarction area. Five vision fields (×100 magnification) were randomly selected from individual HBFP staining sections for measuring the infarction area by the plane method with Leica Qwin analysis software. The parameter was the percentage of infarction area over the total evaluated area, and the average value was then used^32^.

### Quantitative real-time PCR (qRT-PCR)

Total RNA was extracted from the cardiac tissues and cardiomyocytes using TRIzol reagent (Invitrogen, USA) according to the manufacturer’s protocol. RNA concentration was quantified by using NanoDrop (Thermo Fisher Scientific Inc., USA) and then subjected to reverse transcription using a cDNA reverse transcription kit (TaKaRa, Japan) according to the manufacturer’s instructions. A miR-142-3p expression level was detected using Bulge-Loop miRNA qPCR Primer Set (RiboBio, Guangzhou, China) with Takara SYBR supermix Kit (Bio-Rad, Hercules, CA) on ABI 7900HT fast Real-Time PCR System (Applied Biosystems, Foster City, CA). U6 was used as an internal control. For gene expressions, qPCR was performed on ABI 7900HT fast Real-Time PCR System (Applied Biosystems) for 40 cycles using SYBR-Green supermix Kit (Bio-Rad, Hercules, CA). The sequences of primers were listed in Table S1. Glyceraldehyde-3-phosphate dehydrogenase (GAPDH) was used as an internal control. The relative miRNA or gene expression levels were calculated using 2^−ΔΔCT^ method.

### Western blot analysis

Total proteins obtained from the cardiac tissues and cardiomyocytes were separated by 10–15% SDS–PAGE and then electrotransferred onto PVDF membranes (Millipore, Atlanta, GA, US). The membranes were blocked with 5% bovine serum albumin or non-fat milk for 1.5 h at room temperature, followed by incubation at 4 °C overnight with primary antibodies against IRAK-1, NF-κB p65, TNF-α, IL-1β, IL-6, or GAPDH. The primary antibodies specific to IRAK-1 were supplied by Santa Cruz Biotechnology (Santa Cruz, CA, USA). The primary antibodies specific to TNF-α, IL-1β, and IL-6 were supplied by Abcam (Cambridge, MA, USA). The primary antibodies specific to NF-κB p65 and GAPDH were obtained from Cell Signaling Technology (Beverly, MA, USA). After washing with TBS containing 0.1% Tween 20 (TBST) for five times, the membranes were incubated with secondary antibodies conjugated with horseradish peroxidase in TBST for 2 h at room temperature. The signals were detected with an enhanced chemiluminescence detection system (Pierce, Rockford, IL, USA). The protein bands were assessed and quantified by using Image Lab software from Bio-Rad.

### miR-142-3p target gene prediction

Bioinformatics prediction websites TargetScan (http://www.targetscan.org/) and miRBase (http://www.mirbase.org/) were used for target prediction of miR-142-3p. To predict the transcription factor-binding sites in gene promoter regions, the program Alibaba 2.0 (http://gene-regulation.com/pub/programs.html) was used. The National Center for Biotechnology Information’s (https://www.ncbi.nlm.nih.gov/) BLAST program was used for analyzing the sequences of potential target genes.

### Dual luciferase assays

Human embryonic kidney (HEK) 293 cells were seeded at a density of 2 × 10^4^ cells per well in 24-well plates prior to transfection. All transfections were carried out with Lipofectamine 2000 (Invitrogen, Eugene, OR) according to the manufacturer’s instructions. Cells were transfected with pGL3 luciferase expression constructs containing the 3′UTR of IRAK-1 pRL-TK Renilla luciferase vector (Promega, Beijing, China), and miR-142-3p agomiR or NC (GenePharma, Shanghai, China). After transfection for 48 h, luciferase activities were measured using the dual-luciferase reporter assay system (Promega) and normalized to renilla luciferase activity. Meanwhile, we mutated the predicted miR-142-3p-binding site on IRAK-1 3′UTR, and examined whether this mutation could abrogate the decrease in luciferase activity by miR-142-3p agomiR.

### Cell culture and treatment

Primary neonatal rat cardiacmyocytes were isolated and cultured as described previously^33^. The cells were assigned to seven groups, wherein the normal group was treated without any inducer, while 1 μg/ml LPS was added in the remaining six groups to induce cardiomyocyte injury. After 12 h of interference, the cells were collected.

Except for the normal group, the rest six groups were transfected with plasmid separately. The blank group was transfected with no sequences; the NC group was transfected with miR-142-3p NC sequence; the miR-142-3p mimic group was transfected with miR-142-3p agomiR; the miR-142-3p inhibitor group was transfected with miR-142-3p antagomiR; the siRNA-IRAK-1 group was transfected with siRNA-IRAK-1 and the miR-142-3p inhibitor+siRNA-IRAK-1 group was transfected with miR-142-3p antagomiR and siRNA-IRAK-1.

### Plasma miR-142-3p level in STEMI patients undergoing pPCI with no-reflow

Next, we evaluated the clinical relevance between miR-142-3p level in circulation and no-reflow phenomenon in patients with acute STEMI during pPCI. No-reflow is defined as persistence of forward blood flow disorder [thrombolysis in myocardial infarction (TIMI) flow grade ≤ 2] after coronary artery without mechanical obstruction and no significant residual stenosis or dissection^34^. A total of 255 consecutive patients with STEMI undergoing pPCI were enrolled in this study. These patients were divided into two groups according to the occurrence of reflow during pPCI, namely normal-reflow group with 214 cases and no-reflow group with 41 cases. Peripheral venous blood samples were drawn after pPCI for qRT-PCRs detection. In brief, total RNAs were isolated from 200 μl of plasma using a mirVana PARIS isolation kit (Ambion, Austin, TX) according to the manufacturer’s instructions. *Caenorhabditis elegans* miR-39 (cel-miR-39) of 50 pmol/l was added as the spike-in control after adding equal volume of denaturing solution. miR-142-3p was quantified using qRT-PCRs on 7900 HT fast RT-PCR system (Applied Biosystems). This study was conducted according to the principles of the Declaration of Helsinki, and was approved by the Ethics Committee of the First Affiliated Hospital of Guangxi Medical University. All participants provided written informed consent to participate in this study.

### Statistical analysis

Data are presented as means ± standard error of mean (SEM). An independent-Student’s *t*-test was used to compare between two groups. One-way analysis of variance (ANOVA) was performed to compare among three or more groups, followed by Bonferroni’s post-hoc test. The ROC curve analysis was performed with miR-142-3p to distinguish between patients with or without no-reflow. Differences were considered significant at the level of *P* < 0.05.

## Supplementary information

Table S1
