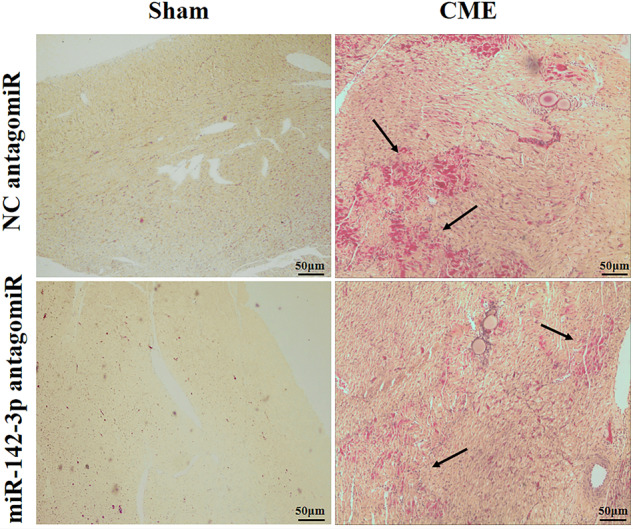# Correction to: The mechanism of miR-142-3p in coronary microembolization-induced myocardiac injury via regulating target gene IRAK-1

**DOI:** 10.1038/s41419-022-04546-w

**Published:** 2022-01-26

**Authors:** Qiang Su, Xiangwei Lv, Ziliang Ye, Yuhan Sun, Binghui Kong, Zhenbai Qin, Lang Li

**Affiliations:** 1 Department of Cardiology, The Affiliated Hospital of Guilin Medical University, 15#, Lequn Road, 541001 Guilin, Guangxi China; 2 Department of Cardiology, The First Affiliated Hospital of Guangxi Medical University, 530021 Nanning, China

**Keywords:** Cardiovascular diseases, Mechanisms of disease

Correction to: *Cell Death Dis* 10.1038/s41419-019-1341-7, published online 25 January 2019

Since online publication of this article the authors noticed there was an error in Fig. 3c. Incorrect images were used for the NC antgomiR and miR-142-3p antagomiR HBFP staining in the Sham group. The figure has been corrected in the online html and PDF. The authors apologize for the error and confirm that these errors do not affect the results or conclusions of the paper.