# Supplementary material for: The mechanism of miR-142-3p in coronary microembolization-induced myocardiac injury via regulating target gene IRAK-1
Source: Cell Death Dis. 2019 Jan 25;10(2):61. doi: 10.1038/s41419-019-1341-7 (PMC6347606; doi:10.1038/s41419-019-1341-7)
Supplement: Supplementary file 1 — Table S1 [file 41419_2019_1341_MOESM1_ESM.docx]

**Table S1. Primer sequences used in this study**

| **name** | **sequence(5’to 3’)** |
| --- | --- |
| **ssc-miR-142-3p** | 5’-TGTAGTGTTTCCTACTTTATGGA-3’ |
| **ssc-miR-142-3p agomir** | 5’-UGUAGUGUUUCCUAUUUAUGG-3’ |
| **ssc-miR-142-3p agomir NC** | 5’-UUCUCCGAACGUGUCACGUTT-3’ |
| **ssc-miR-142-3p antagomir** | 5’-CCAUAAAGUAGGAAACACUACA-3’ |
| **ssc-miR-142-3p antagomir NC** | 5’-CAGUACUUUUGUGUAGUACAA-3’ |
| **U6** | 5’-GCTTCGGCAGCACATATACTAAAAT-3’  5’-CGCTTCACGAATTTGCGTGTCAT-3’ |
| **IRAK-1** | 5’-CCAAACATTGTGGACTTTGC-3’  5’-GGCTGTACCCAGAAGGATGT-3’ |
| **GAPDH** | 5’-GCTTCGGCAGCACATATACTAAAAT-3’  5’-TCTAGACGGCAGGTCAGGTCCAC-3’ |
